# Non-invasive valve implantation with TAVI versus conventional aortic valve replacement

**DOI:** 10.1186/cc12175

**Published:** 2013-03-19

**Authors:** E Trujillo-García, C Joya-Montosa, MJ Delgado-Amaya, E Curiel-Balsera

**Affiliations:** 1 Hospital Regional Carlos Haya, Málaga, Spain

## Introduction

In patients with severe and inoperable aortic stenosis, some trials have proven that TAVI excels standard procedures and has also been proven less cost-effective. We aim at reviewing short-term results after 2 years since the implementation of this technique in our premises.

## Methods

A study of a retrospective cohorts of patients who underwent isolated aortic valve replacement (AVR) by either conventional surgery or TAVI (CoreValve device) from June 2010 to December 2011 at the University Hospital Carlos de Haya (Málaga, Spain). Clinical epidemiologic, complication and short-term outcome variables were registered. Qualitative variables are expressed as percentages, while quantitative variables are expressed as means and SD. Fisher's exact test and Mann-Whitney's U-test were used where necessary (5% maximum error ratio).

## Results

A total number of 27 TAVI and 154 isolated AVR procedures were completed. Intervention typology was chosen according to the recommendations of scientific societies, apart from patients' fulfillment of the anatomic criteria required for percutaneous implant. Mean age was 67 ± 11 years (54% males) in AVR and 80 ± 6 years (44% males) in TAVI (*P <*0.05). The additive EuroSCORE in AVR was 7 ± 2 and 9 ± 2 in TAVI (*P <*0.05). However, 55.6% of the percutaneous-valve patients presented previous coronary-tree alterations with stent implantation, while only 7% of AVR patients showed these alterations (*P <*0.001). ICU mortality in TAVI and AVR patients was 3.7% and 8.2%, respectively (*P *= NS). Regarding complications, 48.1% of TAVI patients showed altered heart rhythm and 33% required a permanent pacemaker. Electrical disorders were observed in 4% of AVR patients, while 1.9% of these patients required a permanent pacemaker (*P <*0.001 for both). Reoperation was necessary in 14.8 and 1.9% of TAVI and AVR patients, respectively (*P <*0.001).

## Conclusion

Even with our limited experience, TAVI patients are observed to be older, to involve higher surgical risk, and to have undergone previous coronary-tree interventions. Although no significant differences were found regarding mortality, a higher rate of complications was observed in TAVI patients. With no short-term differences, a significant rate of postsurgical complications, and a cost-efficiency handicapped technique, analysis of long-term outcomes seems necessary to assess TAVI's advantages over conventional AVR.

